# Preservice laboratory education strengthening enhances sustainable laboratory workforce in Ethiopia

**DOI:** 10.1186/1478-4491-11-56

**Published:** 2013-10-28

**Authors:** Peter N Fonjungo, Yenew Kebede, Wendy Arneson, Derese Tefera, Kedir Yimer, Samuel Kinde, Meseret Alem, Waqtola Cheneke, Habtamu Mitiku, Endale Tadesse, Aster Tsegaye, Thomas Kenyon

**Affiliations:** 1 Division of Global HIV/AIDS, Centers for Disease Control and Prevention (CDC), Addis Ababa, Ethiopia; 2 American Society for Clinical Pathology, Chicago, USA; 3 Department of Medical Laboratory Sciences, College of Health Sciences, School of Allied Health Sciences, Addis Ababa University, Addis Ababa, Ethiopia; 4 College of Medicine and Health Sciences, School of Biomedical and Laboratory Sciences, University of Gondar, Gondar, Ethiopia; 5 Department of Medical Laboratory Sciences and Pathology, College of Public Health and Medical Sciences, Jimma University, Jimma, Ethiopia; 6 Department of Medical Laboratory Sciences, College of Health and Medical Sciences, Haramaya University, Harar, Ethiopia; 7 Department of Medical Laboratory Sciences, College of Medicine and Health Sciences, Hawassa University, Hawassa, Ethiopia; 8 Division of Global HIV/AIDS, Center for Global Health, Centers for Disease Control and Prevention, Atlanta, USA

**Keywords:** Preservice education, PEPFAR, Curriculum, Standardization, Laboratory workforce strengthening, Training, Sustainability

## Abstract

**Background:**

There is a severe healthcare workforce shortage in sub Saharan Africa, which threatens achieving the Millennium Development Goals and attaining an AIDS-free generation. The strength of a healthcare system depends on the skills, competencies, values and availability of its workforce. A well-trained and competent laboratory technologist ensures accurate and reliable results for use in prevention, diagnosis, care and treatment of diseases.

**Methods:**

An assessment of existing preservice education of five medical laboratory schools, followed by remedial intervention and monitoring was conducted. The remedial interventions included 1) standardizing curriculum and implementation; 2) training faculty staff on pedagogical methods and quality management systems; 3) providing teaching materials; and 4) procuring equipment for teaching laboratories to provide practical skills to complement didactic education.

**Results:**

A total of 2,230 undergraduate students from the five universities benefitted from the standardized curriculum. University of Gondar accounted for 252 of 2,230 (11.3%) of the students, Addis Ababa University for 663 (29.7%), Jimma University for 649 (29.1%), Haramaya University for 429 (19.2%) and Hawassa University for 237 (10.6%) of the students. Together the universities graduated 388 and 312 laboratory technologists in 2010/2011 and 2011/2012 academic year, respectively. Practical hands-on training and experience with well-equipped laboratories enhanced and ensured skilled, confident and competent laboratory technologists upon graduation.

**Conclusions:**

Strengthening preservice laboratory education is feasible in resource-limited settings, and emphasizing its merits (ample local capacity, country ownership and sustainability) provides a valuable source of competent laboratory technologists to relieve an overstretched healthcare system.

## Background

The strength of a healthcare system, in addition to physical infrastructure, depends on the skills, competencies, values and availability of its workforce. In sub Saharan Africa, there is a severe shortage and imbalance in its workforce that poses a major threat to achieving the Millennium Development Goals [1]. Due to this critical shortage, healthcare workers are overworked and healthcare gains are at risk of being reversed, especially with the burden of diagnosing and monitoring HIV/AIDS patients on antiretroviral therapy [2]. There is the need to build and maintain an appropriate healthcare workforce to staff the different components of the healthcare system.

The clinical laboratory workforce plays a vital role in healthcare service delivery and has been recognized as one of the six key components for healthcare systems strengthening that would impact and improve the well-being of the community [3]. Properly trained laboratory technologists are needed to obtain reliable results essential for service providers to accurately assess the status of a patient’s health, make accurate diagnoses, design treatment plans and monitor the effectiveness of a specific treatment, as well as for early detection, notification and response to disease outbreaks. Some of the shortages have been linked to a lack of retention strategy and a shortage of appropriate training institutions to build a competent and critical workforce. In some instances, training of healthcare professionals at institutions of higher education has failed to meet current healthcare demands due to a rigid curriculum that fails to evolve, static pedagogy and a lack of adaption to local needs [4]. In sub Saharan Africa, there is a shortage of healthcare professional training schools. For example, the number of degree-granting schools for medical and public healthcare professionals was estimated at 134 and 51, respectively, for a population of 868 million, while in North Africa and the Middle East there were 206 and 46 schools for medical and public healthcare professionals, respectively, for a population of 450 million [4]. In Ethiopia there is a shortage of qualified laboratory technologists among other healthcare workers. In the 2002/2003 Health and Health-related Indicators Report by the Planning and Programming Department of the Ministry of Health (MOH), only 249, 223 and 302 laboratory technologists graduated from five universities with medical laboratory training in 2001, 2002 and 2003, respectively. Additionally, maintaining the quality of laboratory education has become an important challenge in recent years given advances in technology.

The critical shortage of healthcare workers has been further revealed during the HIV/AIDS epidemic in sub Saharan Africa as countries tried to accelerate their response. There was already a shortage of skilled and properly trained healthcare workers, which affected the care and treatment of AIDS patients. Many of the healthcare workers themselves succumbed to AIDS [5,6]. Innovative ways were developed to address shortages of healthcare workers for HIV/AIDS care and treatment delivery services, including in-service training in which existing healthcare workers were trained in specialized skills and areas they lacked. This approach was short-term and involved using multiple implementing partners working with the government to train in-service healthcare workers as needed. Also, the task shifting strategy was emphasized in which a healthcare professional was trained to provide services in more than one specialized area, or the tasks of more highly trained healthcare professionals were shifted to less highly trained healthcare professionals [7-10]. For example, in addition to providing care to AIDS patients, nurses were trained to prescribe and provide ART drugs to AIDS patients [11-13]. For laboratory technologists, in-service training on advanced and complex tests for antiretroviral therapy (ART) monitoring resulted in disruption of the services they normally provided and in an increased task load because of the shortage of laboratory technologists. Because of a high staff turnover, in-service training became quite repetitive, costly and not sustainable.

Preservice education training has an important role in maintaining a steady supply of skilled and knowledgeable healthcare workers to ensure country ownership and sustainability. In the reauthorization of the US President’s Emergency Plan for AIDS Relief (PEPFAR) II, PEPFAR set a target, supported with funding, for training and retention of 140,000 new healthcare workers in recognition that maintaining an appropriate healthcare workforce is a critical component for country ownership, sustainability and overall healthcare system strengthening [14]. The curriculum for preservice education training for laboratory technologists in sub Saharan Africa, in most cases, is not standardized and is heavily skewed towards didactic training with little or no hands-on experience with the laboratory equipment used in the workplace. This is historically due to lack of financial resources to fund and support this equipment in a teaching setting as well as lack of expertise and training of the teaching staff.

The absence of hands-on experience is a major challenge facing preservice education because medical laboratory technology graduates only see this equipment for the first time at healthcare facilities on the first day they have been employed. This may further exacerbate equipment maintenance problems as the newly employed laboratory technologists, if not properly trained, often resort to trial and error with equipment at their new healthcare facility [15]. There is a critical need to build a strong and competent laboratory workforce to properly staff public health laboratories and to provide quality laboratory services. The Field Epidemiology Laboratory Training Program (FELTP) has largely focused on building skills and competency of in-service staff to meet short and medium term skill shortages [16-18].

The goal of this research was to carry out a formative evaluation of the feasibility of strengthening preservice laboratory education in partnership with local universities to ensure a skilled and well trained laboratory workforce for overall healthcare system strengthening.

## Methods

### Study design

The provision of ART monitoring services in laboratory facilities revealed some weaknesses in the laboratory workforce. There was a wide spectrum and knowledge gap among undergraduate laboratory technologists graduating from medical laboratory training universities and joining the workforce. There was limited or no prior exposure with hands-on practicals with ART monitoring equipment, marked differences with students of the same level but different universities, longer duration to orientate and provide practical hands-on training to ensure competency to new graduates in their new facilities with minimal disruption of services. These shortcomings prompted PEPFAR Ethiopia to contact and collaborate with the Department of Medical Laboratory Services, Addis Ababa University (AAU) to improve preservice laboratory education in Ethiopia. Following initial contacts with AAU, there was the need to involve the remaining major public universities. The formative evaluation study describes efforts toward standardization and implementation of curriculum, training of faculty, provision of equipment to complement didactic courses and the impact on preservice education strengthening. The intended outcomes were to successfully develop and implement a standardized curriculum and train faculty students to become competent by practical hands-on experience with ART equipment.

### Assessment

**Figure 1:**
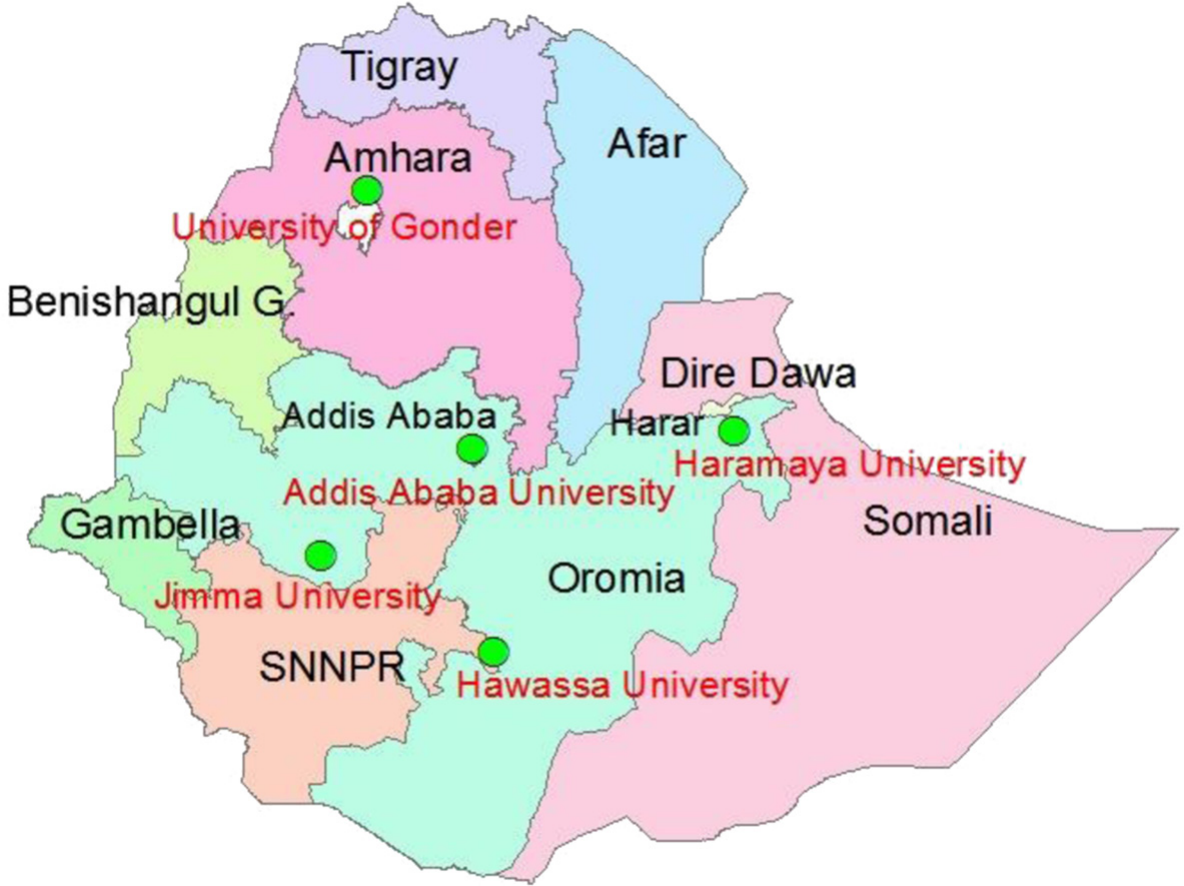
**Location of the five university sites for preservice laboratory education strengthening in Ethiopia.** Circle in green denotes the location of a university and close to each circle is the name of the university in red. The names of the different geographic regions in Ethiopia are shown in black. SNNPR represents Southern Nations, Nationalities and People’s Region.

In 2008, CDC Ethiopia, in collaboration with faculty staff of five universities with medical laboratory technology training schools, conducted a joint assessment of medical laboratory education. The preservice laboratory education strengthening program activity was carried out at all five public universities with medical laboratory technologist training schools throughout Ethiopia (Figure 1). The universities involved were Addis Ababa University, University of Gondar, Jimma University, Hawassa University and Haramaya University. The medical laboratory training schools offered undergraduate (BSc) and postgraduate (MSc) courses. The assessment focused on the curriculum, communications and teaching materials, teaching methods and inventory of laboratory equipment. There were regular meetings held and representatives of all the five universities met to share, discuss and prioritize gaps in training that were revealed by the assessment report. Addis Ababa University medical laboratory school chaired the forum of the five universities and also served as a bridge among CDC Ethiopia, American Society for Clinical Pathology (ASCP) and the other universities. ASCP, one of CDC Ethiopia’s implementing partners also conducted site visits to two of the five university based laboratory schools in 2007 to provide baseline assessment data.

### Intervention

ASCP engaged in different consultative meetings and a scope of work was developed to address some of the training needs that were identified. ASCP then organized three different workshops, and the first workshop was held between March and April 2008 at Addis Ababa University Medical Laboratory School. ASCP was selected because of its wealth of experience in developing curricula and has tailored a curriculum for laboratory technologists training in both the United States and developing countries. In 2010, CDC Ethiopia, together with ASCP, initiated several interventions including standardizing the curriculum, modularization of courses and procurement of equipment at the different laboratory training schools following the assessment. PEPFAR funds were used for procurement of HIV-related equipment, installation reagents and other consumables to strengthen preservice education in the different universities. Faculty staff members were trained on pedagogical skills, research proposal and grant writing skills.

## Results and discussion

### Curriculum standardization

The assessment showed that the curriculum used for teaching at the different university laboratory training schools was not standardized. There were differences in credit hour allocations, course contents and student evaluation methods. In addition, the depths of content materials in each of the subjects varied and were dependent on the instructor’s level of commitment and on the reference materials available. The curriculum did not reflect the strategic objectives of Ethiopia’s integrated national laboratory strategic plan established by MOH/Ethiopian Health and Nutrition Research Institute [19]. The curriculum also needed upgrading in certain areas, including establishing a sequential and logical flow for distributing course content. There were also inadequate and non-standardized teaching materials for lecturers. This hindered interuniversity student transfer or exchange programs, which resulted in inconsistent educational profiles and competency levels.

To resolve these gaps, ASCP consultants organized three workshops to develop and refine a standard curriculum and teaching materials. The curriculum revision mirrored the recommendations by the Ministry of Education (MOE). Participants at the workshop included ASCP consultants, senior faculty and staff, CDC staff, experts from the national reference laboratory, MOH, curriculum experts from MOE, Addis Ababa University and a senior mentor in the medical laboratory field. A total of 98 faculty staff from the five universities participated in the three workshops (Table 1). The revised curriculum included clinical chemistry, hematology, microbiology, parasitology, bacteriology, mycology, virology, immunology, molecular biology, quality management systems and practical training in the use and quality control of health laboratory instrumentation. The newly developed standard curriculum also reflected the needs of the national laboratory strategic plan and MOH needs for public health laboratories. Additionally, teaching contents were developed together with faculty staff and adapted in a modularized format ready for use. The integrated curriculum and teaching content ensured that graduating students from the different schools acquired similar skills and competencies at the BSc degree level offered by their school.

**Table 1:** Number of faculty staff and students trained in 2010/2011 and 2011/2012 academic year

**University**	**Faculty staff training**					**Student practical training**					
	**Training**		**Workshop**			**Hematology analyzer**		**Clinical chemistry analyzer**		**FACScount analyzer**	
	**GWM 2010**	**QMS 2011**	**CRTM 2008**	**TMPS 2009**	**CSR 2010**	**2010/ 2011**	**2011/ 2012**	**2010/ 2011**	**2011/ 2012**	**2010/ 2011**	**2011/2012**
Gondar	7	16	4	5	7	150	155	150	155	150	155
Addis Ababa	12	16	10	7	12	73	164	73	*	73	164
Jimma	7	16	5	6	7	278	301	278	301	278	301
Haramaya	7	16	4	6	7						
Hawassa	7	16	4	7	7						
Total	40	80	27	31	40	501	620	501	456	501	620

Key:

QMS, quality management systems.

GWM, grant writing and management.

CRTM, curriculum revision and teaching methodology.

TMPS, teaching materials preparation and standardization.

CSR, curriculum standardization and refinement.

Note: hematology, clinical chemistry and FACScount analyzers for donation to Haromaya and Hawassa Universities has been completed.

^*^Data not collected due to software malfunction of analyzer and pending maintenance.

In addition, faculty staff of the different universities established a network for sharing of information. The lecturers were also trained on using the new curriculum as well as improving their skills for effective teaching using different methods and classroom lectures. Finally, the lecturers were liaised with ASCP consultants for continuous education through valuable and resourceful website materials.

### Training of faculty and staff and curriculum implementation

ASCP consultants and CDC staff offered intensive training to faculty staff on competitive grant writing/management and quality management systems. ASCP consultants provided pedagogic training to faculty staff, including preparation for lectures, instructional skills, interaction with students, upgrading of lectures to accommodate novel technology and setting and grading of examinations questions. Faculty staff trained in grant writing/management were provided with resources for competitive grants solicitation as well as for quality control and monitoring of performance of laboratory equipment. Experts from CDC trained faculty staff on quality management systems (QMS) including the 12 quality management essentials [20]. A total of 40 and 80 faculty staff from the five universities were trained on grant writing/management and quality management systems in 2010 and 2011, respectively (Table 1). The faculty staff were highly motivated and engaged due to the organization, participatory nature and comprehensive training at the workshop. The faculty staff developed plans to integrate QMS concepts into their classroom and practical teaching, and have implemented QMS at healthcare facilities attached to their universities.

In the 2010/2011 and 2011/2012 academic years, the standardized curriculum and modularized courses were instituted in all five universities for undergraduate students. Together the five universities have a total of 2,230 undergraduate students who benefitted directly from the standardized curriculum, 252 out of 2,230 (11.3%) students from University of Gondar, 663 (29.7%) from Addis Ababa University, 649 (29.1%) from Jimma University, 429 (19.2%) from Haramaya University and 237 (10.6%) from Hawassa University. Together, the five universities graduated 700 laboratory technologists, 388 in the 2010/2011 and 312 in the 2011/2012 academic year, respectively.

### Equipment procurement for practical hands-on training

While students received appropriate didactic training through electronic slide presentations, complementing these lectures with practical hands-on training was limited due to unavailability of appropriate equipment. Previously, it was difficult to assess the competency of the students on different assays without laboratory equipment. For example, prior to the assessment there were no clinical chemistry, hematology and immunophenotyping automated analyzers to complement clinical chemistry, hematology and immunophenotyping courses. Students understandably lacked confidence without practical hands-on training with equipment. The schools sought placements for their students in healthcare facilities with appropriate equipment for hands-on training to complement the courses. This approach was unsuccessful for a variety of reasons. First, the student population was usually large and healthcare facilities regularly providing clinical services were reluctant to engage in placement agreements. In some instances, the placement in healthcare facilities came with conditions. For example, only a certain number of students in the final year were eligible for placement, and the schedules were not compatible with other required courses. This meant a lack of balance and consistency in taught courses and practical hands-on training within and between the different schools. Second, most healthcare facilities simply did not agree to placement agreements. They viewed new trainees who had no prior exposure to these equipment platforms as a potential source of service disruption and equipment malfunction [15]. Admittedly, none of these were realistic reasons for preventing student placements during internship, as these students would eventually graduate and be hired due to the shortage of this critical labor force. In essence, service disruption and equipment malfunction were only temporarily postponed because none of the perceived causes were being addressed.

Funding from PEPFAR was used to procure curriculum-based course manuals, storage drives, computers, LCD projectors, which were donated to all the schools for the lecturers. In 2010, a pilot study was initiated at three universities (Gondar, Addis Ababa, Jimma) by CDC Ethiopia in which three clinical chemistry analyzers, three hematology analyzers, three BD FACScount analyzers, vortex mixers, photometers and uninterrupted power supply (UPS), installation reagents and other laboratory consumables were donated to the universities to establish a strong laboratory for practical hands-on training and experience. To maintain the sustainability of reagent supplies, the university medical laboratory schools reagent request was proposed to be integrated with the university teaching hospital comprehensive reagent procurement. Also, competitive grant solicitation proposals were written by some faculty staff, and funding from potentially awarded grants would enable maintaining reagents for use on the instruments. The consignment of equipment included installation and training at each university of the clinical chemistry, hematology and BD FACScount analyzers. The donated equipment was chosen based on platforms used regularly at healthcare facilities and in keeping with the integrated national laboratory strategic plan, which ensured that graduate students would easily integrate into the healthcare facilities that hire them.

A total of 3,199 students had hands-on training with the analyzers from the three universities in the initial pilot. This number included undergraduate, postgraduate and continuing laboratory education students. In University of Gondar in 2010/2011 academic year, 150 students each had direct practical hands-on training with hematology, chemistry and BD FACScount analyzers respectively (Table 1). Similarly, in 2011/2012 academic year at University of Gondar, 155 students each had training on hematology, chemistry and BD FACScount analyzers. The number of students with hands-on training for the different analyzers for 2010/2011 and 2011/2012 academic years for Addis Ababa and Jimma Universities are shown in Table 1. The equipment has been used for undergraduate and postgraduate students for hands-on training. Observational methodology with corrective actions and laboratory experiments were used to assess skills and competencies of students following practical hands-on training. Additionally, students were assessed in correctly performing assays unsupervised, which counted towards their graduation credits. Postgraduate students have also used the equipment for their MSc thesis projects and lecturers have used it for conducting research grant projects. This has increased the quality of research work and has considerably reduced the universities’ time seeking student placements.

Complementing theoretical lectures with practical hands-on experience on the analyzers had made it easy for students to conceptualize and apply theory. The faculty and staff feedback indicated that the students’ mastery of operating the equipment, using good clinical laboratory practices, performing manufacturer recommended preventive maintenance and analyzing and interpreting results was greatly enhanced and ensured a skilled, confident and competent laboratory workforce upon graduation. These findings are similar to other studies indicating that students’ conceptual and technical understanding of methodologies taught in class was greatly increased with practical hands-on training in the laboratory [21,22].

To ensure sustainability and the use of the equipment, the universities were committed to providing reagents for use in training. In addition, the schools have engaged service maintenance contracts with equipment vendors or their representatives to attend to any maintenance needs. Despite the commitment undertaken by universities to maintain equipment, equipment maintenance still remains a challenge [15]. Due to software damage of the chemistry analyzers and pending maintenance at Addis Ababa University, students could not be trained on this platform in 2011/2012 (Table 1). The success of the pilot of equipment donated to the three universities has enabled the procurement of similar equipment for donation to the remaining two universities (Haramaya and Hawassa).

### Advantages and limitations of study

One of the major advantages of the study was the development of a standardized curriculum that was implemented in five universities that enabled and maintained consistency across the various universities. Additionally, the provision of ART monitoring equipment for practical hands on training that complemented didactic lessons.

Limitations of this study include the absence of a follow-up survey of graduated technologists, their employers, to assess the performance of the technologists in their new workplace. The findings of such a survey would have provided a more accurate measure of the effectiveness of the revised curriculum and practical hands-on training by new graduates. Nevertheless, it is noteworthy that the standardized curriculum across the five universities and the introduction of practical hands-on training on equipment were used for academic evaluation and scoring of the students prior to recommendation for graduation.

## Conclusions

Preservice laboratory education strengthening is feasible with situational analysis, careful planning, involvement of appropriate stakeholders and appropriate intervention including procurement of appropriate instruments. The development of a standardized curriculum, integration of teaching modules and complementing students training with hands-on training on equipment greatly enhanced the skills, competency and confidence of the students and ensures maintaining a valuable source of quality laboratory staff. CDC Ethiopia, working directly with indigenous local universities, ensures local capacity building and sustainability. Continued advocacy and partnerships is key to implementing successful preservice laboratory education strengthening programs.

## Abbreviations

ART: Antiretroviral therapy; BD: Becton Dickinson; PEPFAR: US president’s emergency plan for AIDS relief; QMS: Quality management system.

## Competing interests

The authors have no conflicts of interest to disclose. The findings and conclusions in this report are those of the authors and do not necessarily represent the views of the Centers for Disease Control and Prevention.

## Authors’ contributions

PNF, YK, AT and TK conceived the study and design. PNF wrote the first draft. DT, WA, KY, SK, MA, WC, HM, ET, and AT participated in implementing and monitoring the study as well as collecting data, and TK provided general oversight and guidance in implementing the study as well as critically reviewing and editing the manuscript. All authors participated in the write-up, review, feedback and approval of the manuscript.
